# Supplementary figures and images for: A new multiplex SARS-CoV-2 antigen microarray showed correlation of IgG, IgA, and IgM antibodies from patients with COVID-19 disease severity and maintenance of relative IgA and IgM antigen binding over time
Source: PLoS One. 2023 Mar 30;18(3):e0283537. doi: 10.1371/journal.pone.0283537 (PMC10062637; doi:10.1371/journal.pone.0283537)

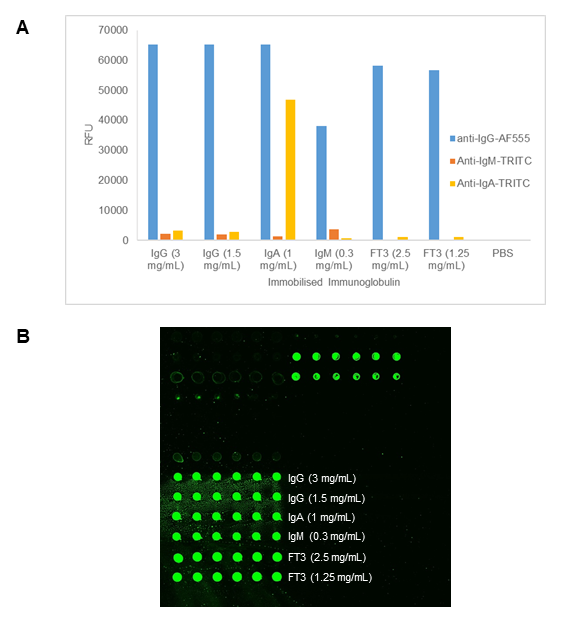

Supplement: S1 Fig — IgG was printed at 3 and 1.5 mg/mL, IgA at 1 mg/mL and IgM at 0.3 mg/mL and the flow through (FT3) at 2.5 and 1.25 mg/mL. (A) Bar chart representing the binding intensity of the fluorescently-labelled anti-isotype antibodies binding to immobilised IgG, IgA and IgM, incubated at 1, 2, and 1 μg/mL, respectively. (B) Scanned image of a subarray depicting the printed serum IgG, IgA, IgM and Ig-depleted FT3 incubated with fluorescently labelled anti-IgG antibody. The cross-reactivity of the anti-IgG antibody for IgA and IgM can be clearly observed and remaining IgGs were also detected in the Ig-depleted flow through FT3. (TIF) [file pone.0283537.s001.tif]

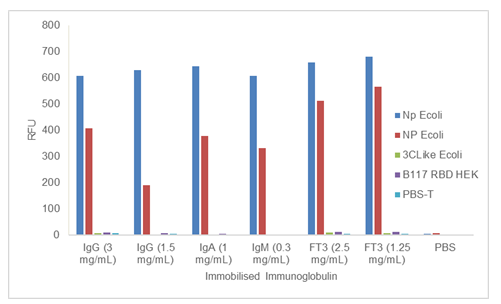

Supplement: S2 Fig — Bar chart representing the binding intensity of His-tagged recombinant SARS-CoV-2 protein antigens to printed serum antibody isotypes purified from one SARS-CoV-2-positive patient (severe disease) serum sample. The serum antibody isotype microarray slides were incubated with a panel of antigens: NP Ecoli at 67 and 27 μg/mL, 3CLike Ecoli at 70 μg/mL and B117 RBD HEK at 17 μg/mL. Antigen binding was detected by incubation with fluorescently-labelled anti-His antibody at 1 μg/mL. (TIF) [file pone.0283537.s002.tif]

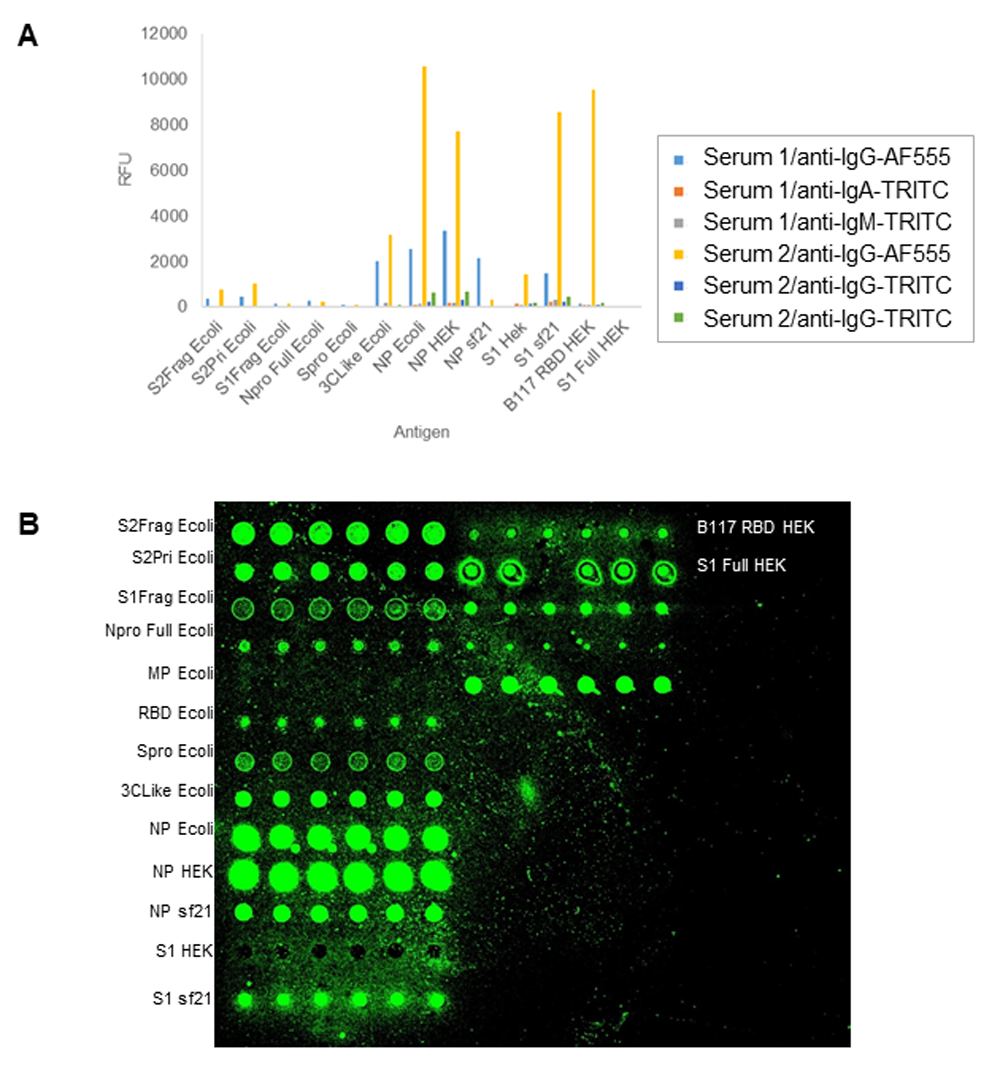

Supplement: S3 Fig — (A) Serum antibody isotype binding to SARS-CoV-2 antigens on microarray detected by either tetramethylrhodamine- (TRITC-) or AlexaFluor® 555- (AF555-)labelled anti-isotype antibodies. (B) Representative image of a subarray from an antigen microarray slide incubated with SARS-CoV-2-positive patient serum followed by detection with anti-IgG-AF555. (TIF) [file pone.0283537.s003.tif]

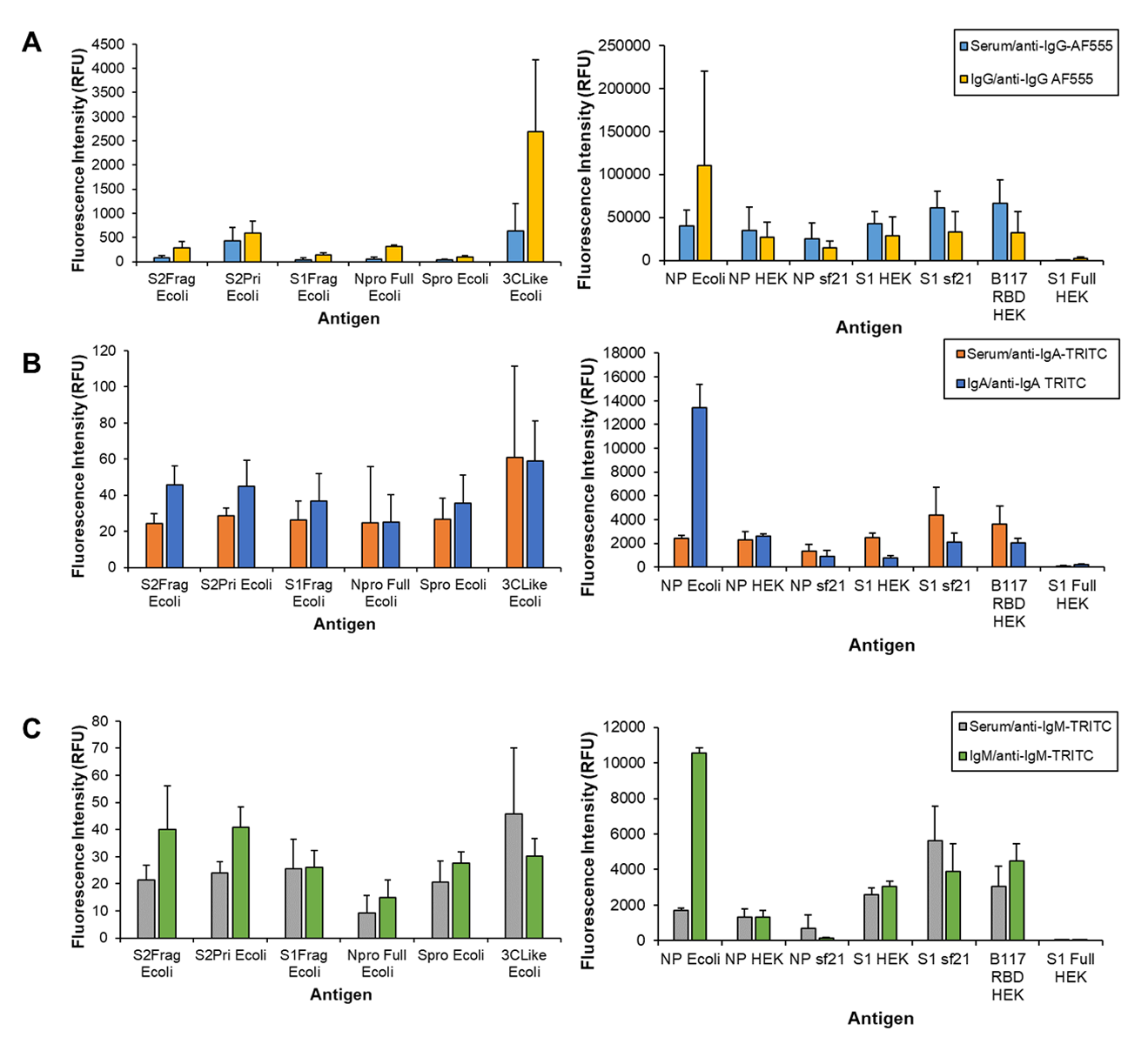

Supplement: S4 Fig — Binding comparisons of (A) serum and purified serum IgG, (B) serum and purified IgA, and (C) serum and purified IgM. Comparisons of serum versus purified antibody isotype detection are shown across two charts to allow visualisation of lower serum antibody isotype binding to certain antigens. (TIF) [file pone.0283537.s004.tif]

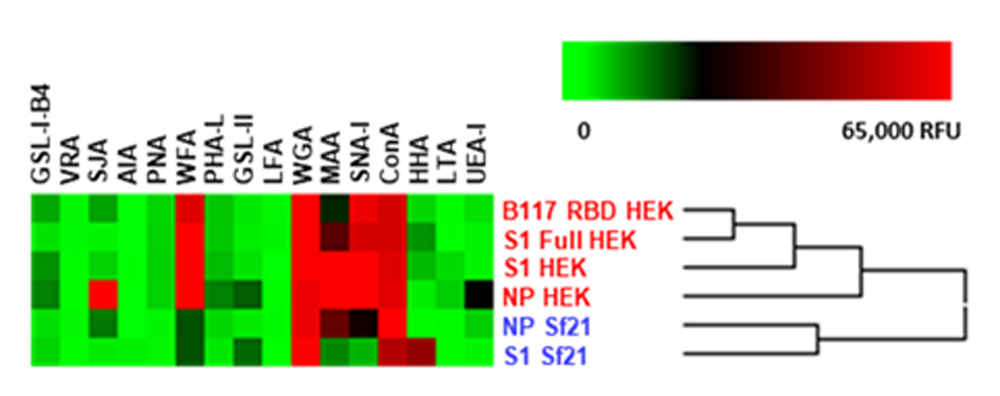

Supplement: S5 Fig — Unsupervised clustering of binding intensities of a panel of fluorescently-labelled lectins to recombinant SARS-CoV-2 proteins printed on the antigen microarray. The lectin LFA was non-functional in this format and was disregarded. Normalised data were subjected to unsupervised, Euclidean distance, complete linkage clustering. (TIF) [file pone.0283537.s005.tif]

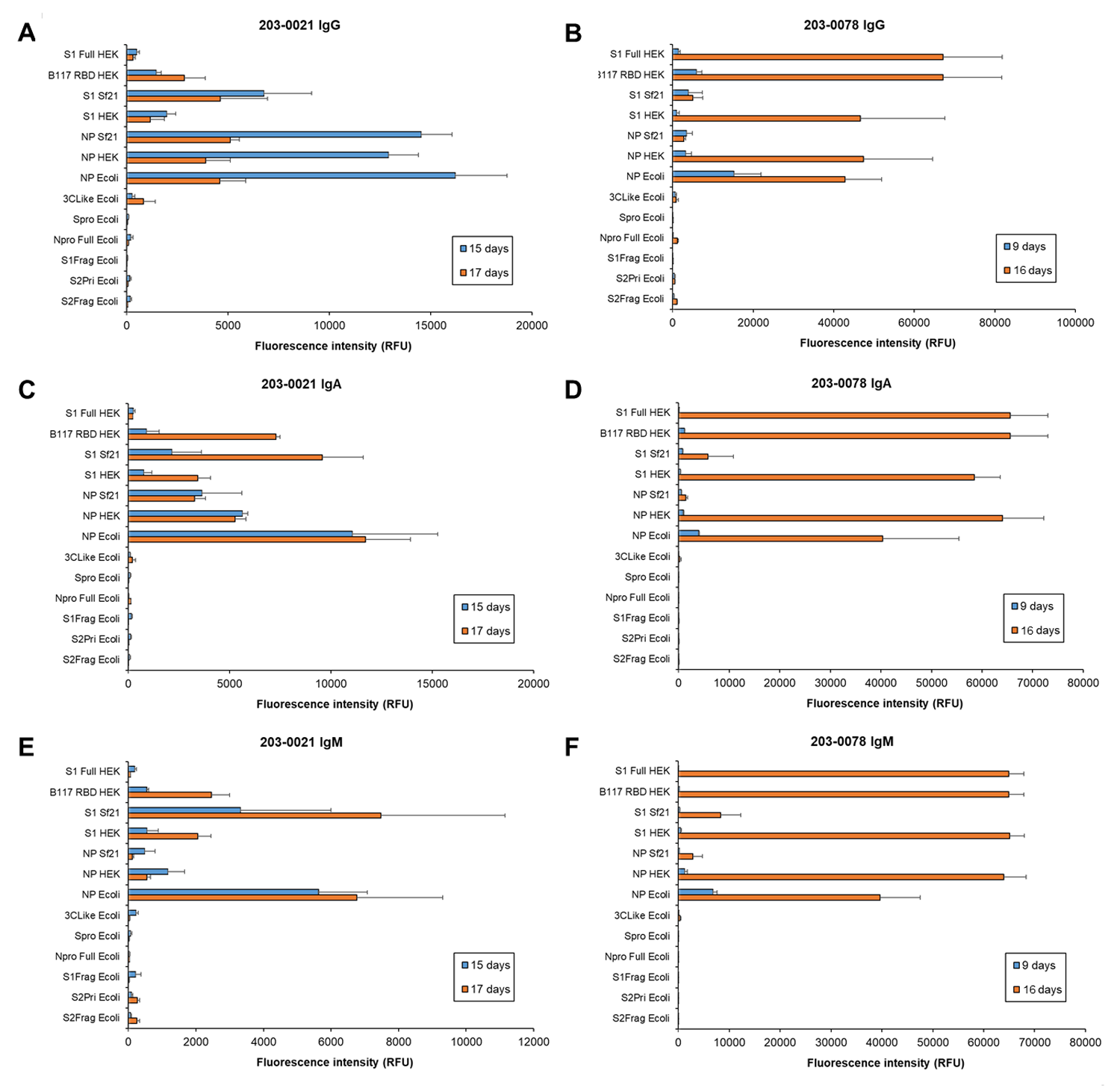

Supplement: S6 Fig — Bar charts represent binding intensity data for serum (A,B) IgG, (C,D) IgA, and (E,F) IgM binding to SARS-CoV-2 antigens for patient 203–0021 (A,C,D; M, 65 years) and 203–0078 (B,D,F; F, 35 years) at 15 and 17 days, and 9 and 16 days post-first symptom onset, respectively. (TIF) [file pone.0283537.s006.tif]

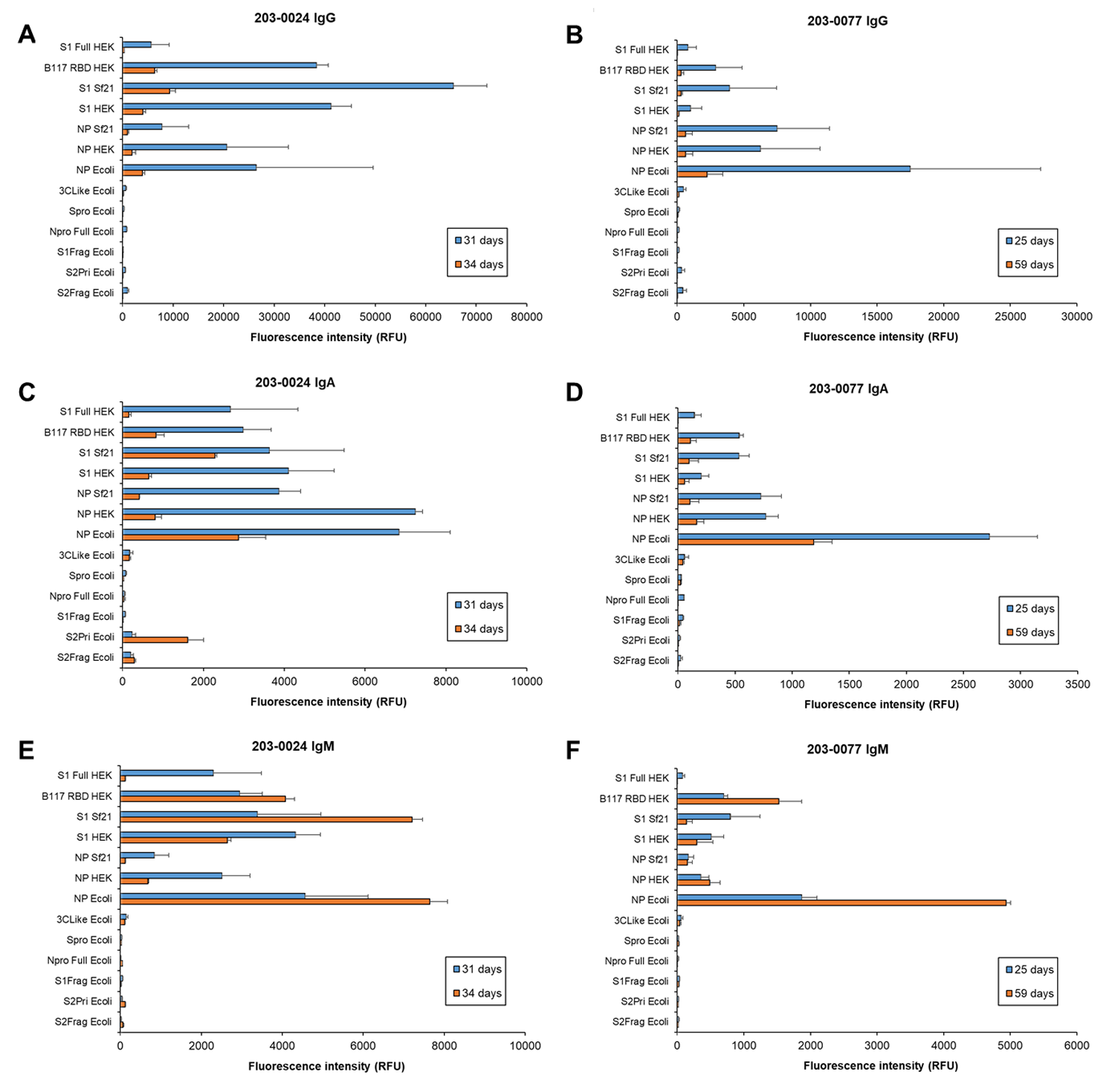

Supplement: S7 Fig — Bar charts represent binding intensity data for serum (A,B) IgG, (C,D) IgA, and (E,F) IgM binding to SARS-CoV-2 antigens for patient 203–0024 (A,C,D; severe, F, 66 years) and 203–0078 (B,D,F; mild, M, 62 years) at 31 and 34 days, and 25 and 59 days post-first symptom onset, respectively. (TIF) [file pone.0283537.s007.tif]

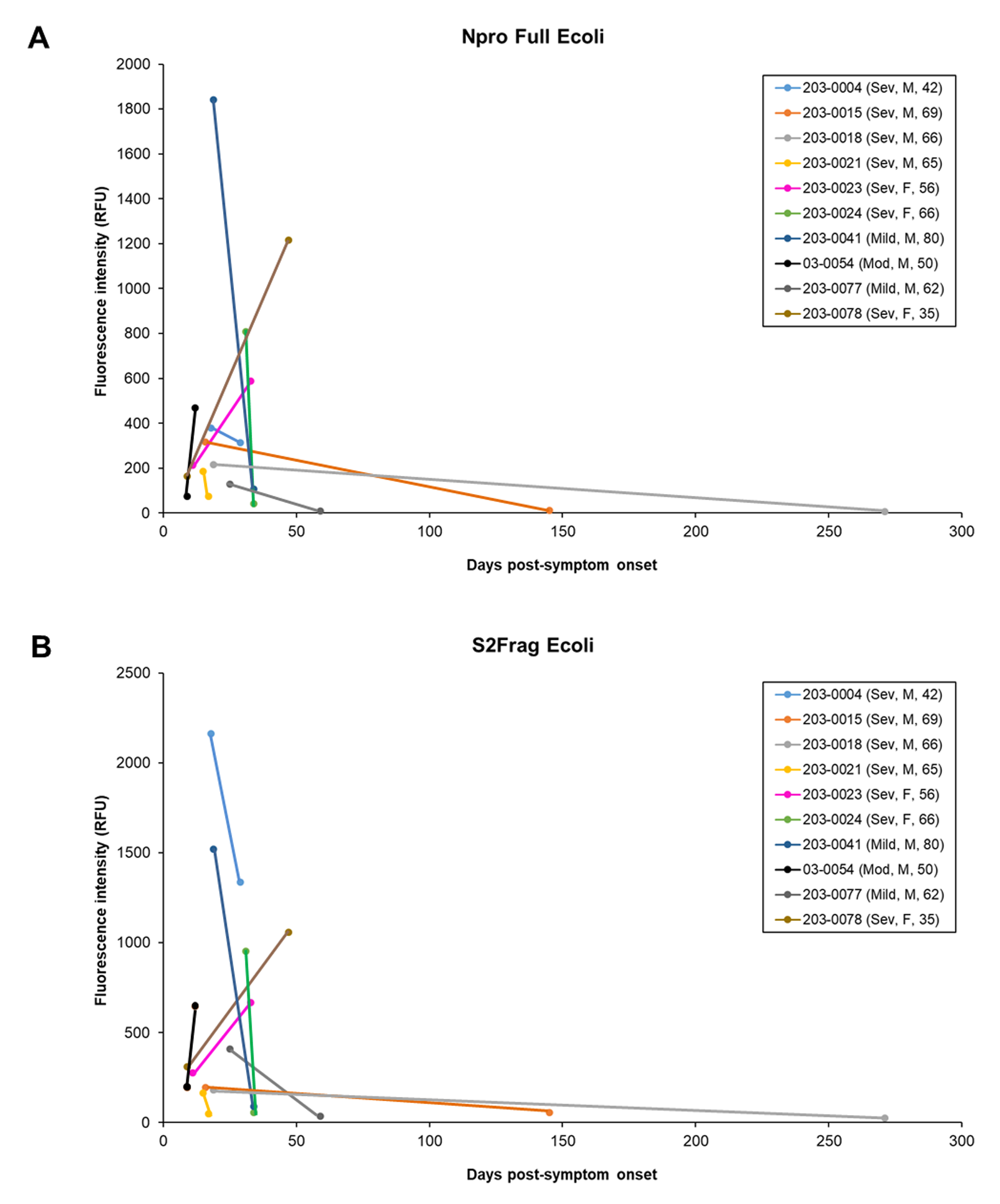

Supplement: S8 Fig. Variation in IgG binding to (A) Npro Full Ecoli and (B) S2Frag Ecoli for a subset of patient samples — (TIF) [file pone.0283537.s008.tif]
