# Supplementary material for: A new multiplex SARS-CoV-2 antigen microarray showed correlation of IgG, IgA, and IgM antibodies from patients with COVID-19 disease severity and maintenance of relative IgA and IgM antigen binding over time
Source: PLoS One. 2023 Mar 30;18(3):e0283537. doi: 10.1371/journal.pone.0283537 (PMC10062637; doi:10.1371/journal.pone.0283537)
Supplement: S1 Table — Including the Ag code, approximate print concentration (mg/mL), molecular mass observed by SDS-PAGE, expression system, protein sequence expressed, buffer that the protein was supplied in, supplier, and catalogue number. MPL, Molecular Parasitology Lab; RB, RayBiotech; R&D, R&D Systems. (PDF) [file pone.0283537.s009.pdf]

Table S1. Recombinantly expressed SARS-CoV-2 proteins used to construct the antigen (Ag) microarray, the Ag code, approximate print concentration (mg/mL), molecular mass observed by SDS-PAGE, expression system, protein sequence expressed, buffer that the protein was supplied in, supplier, and catalogue number. MPL, Molecular Parasitology Lab; RB, RayBiotech; R&D, R&D Systems.

Ag code	Protein Ag	Print conc	Mr (kDa)	Expression	Sequence	Buffer	Supplier	Cat. no.
Npro Full Ecoli	Nucleocapsid protein, full length	0.06	50	*E. coli*	2-1269	PBS + 500 mM imidazole	MPL	-
3CLike Ecoli	3C-like protease	0.24	34	*E. coli*	1-306	PBS	MPL	-
Spro Ecoli	Spike protein, full length	0.12	135	*E. coli*	10-1282	Potassium phosphate + 0.1% Sarkosyl, pH 7.4	MPL	-
S1Frag Ecoli	Spike protein fragment 1	0.15	75	*E. coli*	10-686	Potassium phosphate + 0.1% Sarkosyl, pH 7.4	MPL	-
S2Frag Ecoli	Spike protein fragment 2	0.06	54	*E. coli*	687-1283	Potassium phosphate + 0.1% Sarkosyl, pH 7.4	MPL	-
S2Pri Ecoli	Spike protein fragment 2 prime region	0.01	38	*E. coli*	816-1283	PBS+0.05% Sarkosyl	MPL	-
NP Ecoli	Nucleocapsid protein	0.95	50	*E. coli*	Met1-Ala419	PBS	RB	230-01104
NP HEK	Nucleocapsid protein	0.6	50-60	HEK293	Met1-Ala419	PBS	RB	230-30164
NP Sf21	Nucleocapsid protein	0.5	44-53	Sf21	Met1-Ala419	PBS	R&D	10474-CV
S1 HEK	S1 subunit protein	0.5	106-121	HEK293	Val16-Pro681	PBS with trehalose	R&D	10569-CV
S1 Sf21	S1 subunit protein	0.2	78-92	Sf21	Val16-Pro681	PBS with trehalose	R&D	10522-CV
B117 RBD HEK	B.1.1.7 receptor binding domain	0.25	34-38	HEK293	Arg319-Phe541	PBS with trehalose	R&D	10730-CV
S1 Full HEK	S1 subunit protein, full length	0.45	120	HEK293	Val16-Gln690	PBS	RB	230-30161
MP Ecoli	Membrane glycoprotein, C-terminal	0.17	15	*E. coli*	Arg101-Gln222	PBS + 200 mM imidazole	RB	230-01124
