# Supplementary material for: A new multiplex SARS-CoV-2 antigen microarray showed correlation of IgG, IgA, and IgM antibodies from patients with COVID-19 disease severity and maintenance of relative IgA and IgM antigen binding over time
Source: PLoS One. 2023 Mar 30;18(3):e0283537. doi: 10.1371/journal.pone.0283537 (PMC10062637; doi:10.1371/journal.pone.0283537)
Supplement: S2 Table — Including their binding specificities and concentrations used (μg/mL). (PDF) [file pone.0283537.s010.pdf]

Table S2. Lectins used for recombinant SARS-CoV-2 proteins glycoprofiling, their binding specificities and concentrations used (μg/mL).

Lectin	Abbreviation	Major binding specificity	Conc.
Griffonia simplicifolia lectin	GSL-I-B4	Terminal α-linked Gal	10
Vigna radiate lectin	VRA	Terminal α-linked Gal	10
Artocarpus integrifolia agglutinin	AIA	Gal-β-(1→3)-GalNAc (T-antigen), β-(1→6)-linked Gal, sialylation independent	15
Arachis hypogaea lectin	PNA	Gal-β-(1→3)-GalNAc (T-antigen) >GalNAc>Lac>Gal, terminal β-Gal)	10
Sophora japonica lectin	SJA	β-linked GalNAc>Gal	10
Wisteria floribunda agglutinin	WFA	GalNAc (GalNAc-α-(1→6)-Gal>GalNAc-α-(1→3)-R GalNAc (Forsmann antigen) >GalNAc>>Lac>Gal, chondroitin sulfate	10
Phaseolus vulgaris lectin	PHA-L	tri-, tetra-antennary β-Gal/Gal-β-(1→4)-GlcNAc	10
Griffonia simplicifolia lectin	GSL-II	GlcNAc	10
Triticum vulgaris lectin	WGA	NeuAc/GlcNAc	10
Limax flavus lectin	LFA	NeuAc/NeuGc	10
Maackia amurensis agglutinin	MAA	Neu-α-(2→3)-Gal = Gal-3-SO_4 >Lac	15
Sambucus nigra agglutinin I	SNA-I	Neu-α-(2→6)-Gal(NAc) >Lac, GalNAc >Gal	10
Canavalia ensiformis (jack bean lectin)	Con A	α-linked Man>Glc>GlcNAc	10
Hippeastrum hybrid lectin	HHA	Man-α-(1,3)-Man-α-(1,6)-	10
Lotus tetragonolobus lotus lectin	LTA	α-(1→3)-linked Fuc	10
Ulex europaeus agglutinin I	UEA-I	α-(1→2)-linked Fuc, H type 2 antigen	10
