# Supplementary material for: A new multiplex SARS-CoV-2 antigen microarray showed correlation of IgG, IgA, and IgM antibodies from patients with COVID-19 disease severity and maintenance of relative IgA and IgM antigen binding over time
Source: PLoS One. 2023 Mar 30;18(3):e0283537. doi: 10.1371/journal.pone.0283537 (PMC10062637; doi:10.1371/journal.pone.0283537)
Supplement: S4 Table — Significance of correlation analysis for first sampling point serum IgG samples binding to antigens with low intensity (3CLike Ecoli, Npro Full Ecoli, S1 Frag Ecoli, S2 Frag Ecoli, S2Pri Ecoli, and Spro Ecoli) compared to non-COVID-19 samples (NC). Bold p value indicates significant correlation. (PDF) [file pone.0283537.s012.pdf]

Table S4. Significance of correlation analysis for first sampling point serum IgG samples binding to antigens with low intensity (3CLike Ecoli, Npro Full Ecoli, S1 Frag Ecoli, S2 Frag Ecoli, S2Pri Ecoli, and Spro Ecoli) compared to non-COVID-19 samples (NC). Bold *p* value indicates significant correlation.

	**IgG**		
Predictors	*Estimates*	*CI*	*p*
(Intercept)	77.83	53.99 – 101.67	**<0.001**
COVID.19 [Mild]	-36.13	-73.61 – 1.35	0.058
COVID.19 [Moderate]	53.45	17.32 – 89.59	**0.005**
COVID.19 [Severe]	-15.83	-50.00 – 18.34	0.352
Antigen [Npro Full Ecoli]	-39.05	-64.36 – -13.74	**0.003**
Antigen [S1Frag Ecoli]	-52.59	-77.90 – -27.28	**<0.001**
Antigen [S2Frag Ecoli]	-57.60	-82.91 – -32.29	**<0.001**
Antigen [S2Pri Ecoli]	-52.38	-77.69 – -27.07	**<0.001**
Antigen [Spro Ecoli]	-56.34	-81.65 – -31.03	**<0.001**
COVID.19 [Mild] * Antigen [Npro Full Ecoli]	19.82	-18.44 – 58.08	0.309
COVID.19 [Moderate] *Antigen [Npro Full Ecoli]	-71.14	-108.03 – -34.24	**<0.001**
COVID.19 [Severe] *Antigen [Npro Full Ecoli]	15.05	-19.83 – 49.94	0.397
COVID.19 [Mild] * Antigen [S1Frag Ecoli]	80.40	42.14 – 118.67	**<0.001**
COVID.19 [Moderate] *Antigen [S1Frag Ecoli]	-52.75	-89.64 – -15.85	**0.005**
COVID.19 [Severe] *Antigen [S1Frag Ecoli]	44.99	10.10 – 79.88	**0.012**
COVID.19 [Mild] * Antigen [S2Frag Ecoli]	76.03	37.77 – 114.30	**<0.001**
COVID.19 [Moderate] *Antigen [S2Frag Ecoli]	-45.17	-82.07 – -8.28	**0.017**
COVID.19 [Severe] *Antigen [S2Frag Ecoli]	58.58	23.69 – 93.46	**0.001**
COVID.19 [Mild] * Antigen [S2Pri Ecoli]	63.15	24.88 – 101.41	**0.001**
COVID.19 [Moderate] *Antigen [S2Pri Ecoli]	-56.01	-92.91 – -19.12	**0.003**
COVID.19 [Severe] *Antigen [S2Pri Ecoli]	59.34	24.45 – 94.23	**0.001**
COVID.19 [Mild] * Antigen [Spro Ecoli]	63.97	25.71 – 102.24	**0.001**
COVID.19 [Moderate] *Antigen [Spro Ecoli]	-44.13	-81.02 – -7.23	**0.019**
COVID.19 [Severe] *Antigen [Spro Ecoli]	42.76	7.87 – 77.64	**0.016**
N_{id}	34		
Observations	612		
