# Supplementary material for: A new multiplex SARS-CoV-2 antigen microarray showed correlation of IgG, IgA, and IgM antibodies from patients with COVID-19 disease severity and maintenance of relative IgA and IgM antigen binding over time
Source: PLoS One. 2023 Mar 30;18(3):e0283537. doi: 10.1371/journal.pone.0283537 (PMC10062637; doi:10.1371/journal.pone.0283537)
Supplement: S5 Table — Significance of correlation analysis for first sampling point serum IgG samples binding to antigens with high intensity (B117 RBD HEK, NP Ecoli, NP HEK, NP Sf21, S1 Full HEK, S1 HEK, and S1 Sf21) compared to non-COVID-19 samples (NC). Bold p value indicates significant correlation. (PDF) [file pone.0283537.s013.pdf]

Table S5. Significance of correlation analysis for first sampling point serum IgG samples binding to antigens with high intensity (B117 RBD HEK, NP Ecoli, NP HEK, NP Sf21, S1 Full HEK, S1 HEK, and S1 Sf21) compared to non-COVID-19 samples (NC). Bold *p* value indicates significant correlation.

	IgG		
Predictors	*Estimates*	*CI*	*p*
(Intercept)	1229.48	431.16 – 2027.79	**0.003**
COVID.19 [Mild]	-589.85	-1845.15 – 665.45	0.345
COVID.19 [Moderate]	174.53	-1035.83 – 1384.90	0.770
COVID.19 [Severe]	245.56	-898.94 – 1390.06	0.664
Antigen [NP Ecoli]	4531.91	3914.78 – 5149.04	**<0.001**
Antigen [NP HEK]	-201.15	-818.28 – 415.99	0.522
Antigen [NP Sf21]	-710.09	-1327.22 – -92.96	**0.024**
Antigen [S1 Full HEK]	-1041.49	-1658.62 – -424.36	**0.001**
Antigen [S1 Hek]	-994.09	-1611.22 – -376.95	**0.002**
Antigen [S1 Sf21]	-410.67	-1027.80 – 206.46	0.192
COVID.19 [Mild] *Antigen [NP Ecoli]	-2010.93	-2943.94 – -1077.91	**<0.001**
COVID.19 [Moderate] *Antigen [NP Ecoli]	-1984.75	-2884.36 – -1085.13	**<0.001**
COVID.19 [Severe] *Antigen [NP Ecoli]	-399.88	-1250.54 – 450.78	0.356
COVID.19 [Mild] * Antigen [NP HEK]	432.73	-500.29 – 1365.74	0.363
COVID.19 [Moderate] *Antigen [NP HEK]	-137.27	-1036.88 – 762.35	0.765
COVID.19 [Severe] *Antigen [NP HEK]	1381.22	530.56 – 2231.88	**0.001**
COVID.19 [Mild] * Antigen [NP Sf21]	516.01	-417.01 – 1449.02	0.278
COVID.19 [Moderate] *Antigen [NP Sf21]	301.42	-598.20 – 1201.04	0.511
COVID.19 [Severe] *Antigen [NP Sf21]	684.68	-165.98 – 1535.34	0.114
COVID.19 [Mild] * Antigen [S1 Full HEK]	823.52	-109.49 – 1756.54	0.084
COVID.19 [Moderate] *Antigen [S1 Full HEK]	-304.15	-1203.77 – 595.46	0.507
COVID.19 [Severe] *Antigen [S1 Full HEK]	-55.03	-905.69 – 795.62	0.899
COVID.19 [Mild] * Antigen [S1 HEK]	1081.81	148.80 – 2014.83	**0.023**
COVID.19 [Moderate] *Antigen [S1 HEK]	94.92	-804.70 – 994.53	0.836
COVID.19 [Severe] *Antigen [S1 HEK]	571.77	-278.89 – 1422.42	0.187
COVID.19 [Mild] * Antigen [S1 Sf21]	563.84	-369.18 – 1496.85	0.236
COVID.19 [Moderate] *Antigen [S1 Sf21]	1187.56	287.95 – 2087.18	**0.010**
COVID.19 [Severe] *Antigen [S1 Sf21]	1536.96	686.30 – 2387.62	**<0.001**
N_{id}	34		
Observations	714		
