# Supplementary material for: A new multiplex SARS-CoV-2 antigen microarray showed correlation of IgG, IgA, and IgM antibodies from patients with COVID-19 disease severity and maintenance of relative IgA and IgM antigen binding over time
Source: PLoS One. 2023 Mar 30;18(3):e0283537. doi: 10.1371/journal.pone.0283537 (PMC10062637; doi:10.1371/journal.pone.0283537)
Supplement: S6 Table — Significance of correlation analysis for first sampling point serum IgA samples binding to antigens with low intensity (3CLike Ecoli, Npro Full Ecoli, S1 Frag Ecoli, S2 Frag Ecoli, S2Pri Ecoli, and Spro Ecoli) compared to non-COVID-19 (NC) samples. Bold p value indicates significant correlation. (PDF) [file pone.0283537.s014.pdf]

Table S6. Significance of correlation analysis for first sampling point serum IgA samples binding to antigens with low intensity (3CLike Ecoli, Npro Full Ecoli, S1 Frag Ecoli, S2 Frag Ecoli, S2Pri Ecoli, and Spro Ecoli) compared to non-COVID-19 (NC) samples. Bold *p* value indicates significant correlation.

	IgA		
Predictors	*Estimates*	*CI*	*p*
(Intercept)	483.97	192.70 – 775.23	**0.001**
COVID.19 [Mild]	102.62	-355.23 – 560.47	0.650
COVID.19 [Moderate]	995.78	554.32 – 1437.24	**<0.001**
COVID.19 [Severe]	0.29	-417.15 – 417.72	0.999
Antigen [Npro Full Ecoli]	-372.02	-682.44 – -61.59	**0.019**
Antigen [S1Frag Ecoli]	-431.12	-741.55 – -120.70	**0.007**
Antigen [S2Frag Ecoli]	-287.50	-597.92 – 22.93	0.069
Antigen [S2Pri Ecoli]	-283.09	-593.52 – 27.33	0.074
Antigen [Spro Ecoli]	-443.22	-753.65 – -132.80	**0.005**
COVID.19 [Mild] * Antigen [Npro Full Ecoli]	206.66	-262.66 – 675.98	0.387
COVID.19 [Moderate] *Antigen [Npro Full Ecoli]	-905.28	-1357.80 – -452.76	**<0.001**
COVID.19 [Severe] *Antigen [Npro Full Ecoli]	170.46	-257.43 – 598.36	0.434
COVID.19 [Mild] * Antigen [S1Frag Ecoli]	-67.87	-537.19 – 401.45	0.776
COVID.19 [Moderate] *Antigen [S1Frag Ecoli]	-916.57	-1369.09 – -464.05	**<0.001**
COVID.19 [Severe] *Antigen [S1Frag Ecoli]	16.14	-411.75 – 444.03	0.941
COVID.19 [Mild] * Antigen [S2Frag Ecoli]	176.54	-292.78 – 645.86	0.460
COVID.19 [Moderate] *Antigen [S2Frag Ecoli]	-785.90	-1238.42 – -333.38	**0.001**
COVID.19 [Severe] *Antigen [S2Frag Ecoli]	282.46	-145.43 – 710.35	0.195
COVID.19 [Mild] *Antigen [S2Pri Ecoli]	8.25	-461.07 – 477.57	0.972
COVID.19 [Moderate] *Antigen [S2Pri Ecoli]	-850.66	-1303.18 – -398.15	**<0.001**
COVID.19 [Severe] *Antigen [S2Pri Ecoli]	505.82	77.93 – 933.71	**0.021**
COVID.19 [Mild] *Antigen [Spro Ecoli]	-18.40	-487.72 – 450.92	0.939
COVID.19 [Moderate] *Antigen [Spro Ecoli]	-866.72	-1319.24 – -414.20	**<0.001**
COVID.19 [Severe] *Antigen [Spro Ecoli]	78.89	-349.00 – 506.78	0.717
N_{id}	34		
Observations	612		
