# Supplementary material for: A new multiplex SARS-CoV-2 antigen microarray showed correlation of IgG, IgA, and IgM antibodies from patients with COVID-19 disease severity and maintenance of relative IgA and IgM antigen binding over time
Source: PLoS One. 2023 Mar 30;18(3):e0283537. doi: 10.1371/journal.pone.0283537 (PMC10062637; doi:10.1371/journal.pone.0283537)
Supplement: S7 Table — Significance of correlation analysis for first sampling point serum IgA samples binding to antigens with high intensity (B117 RBD HEK, NP Ecoli, NP HEK, NP Sf21, S1 Full HEK, S1 HEK, and S1 Sf21) compared to non-COVID-19 samples (NC). Bold p value indicates significant correlation. (PDF) [file pone.0283537.s015.pdf]

Table S7. Significance of correlation analysis for first sampling point serum IgA samples binding to antigens with high intensity (B117 RBD HEK, NP Ecoli, NP HEK, NP Sf21, S1 Full HEK, S1 HEK, and S1 Sf21) compared to non-COVID-19 samples (NC). Bold *p* value indicates significant correlation.

		**IgA**	
Predictors	*Estimates*	*CI*	*p*
(Intercept)	2923.08	-3469.64 – 9315.81	0.370
COVID.19 [Mild]	1262.03	-8790.16 – 11314.22	0.799
COVID.19 [Moderate]	8141.82	-1550.54 – 17834.17	0.097
COVID.19 [Severe]	15722.54	6557.67 – 24887.42	**0.001**
Antigen [NP Ecoli]	9101.42	4381.07 – 13821.77	**<0.001**
Antigen [NP HEK]	804.24	-3916.11 – 5524.59	0.738
Antigen [NP Sf21]	-744.45	-5464.80 – 3975.90	0.757
Antigen [S1 Full HEK]	-2210.84	-6931.19 – 2509.51	0.358
Antigen [S1 Hek]	-2322.92	-7043.28 – 2397.43	0.334
Antigen [S1 Sf21]	-568.70	-5289.05 – 4151.65	0.813
COVID.19 [Mild] * Antigen [NP Ecoli]	1777.22	-5359.28 – 8913.71	0.625
COVID.19 [Moderate] *Antigen [NP Ecoli]	6631.45	-249.58 – 13512.49	0.059
COVID.19 [Severe] *Antigen [NP Ecoli]	2805.48	-3701.08 – 9312.03	0.397
COVID.19 [Mild] * Antigen [NP HEK]	2800.65	-4335.85 – 9937.15	0.441
COVID.19 [Moderate] *Antigen [NP HEK]	2122.99	-4758.05 – 9004.02	0.545
COVID.19 [Severe] *Antigen [NP HEK]	-4032.48	-10539.03 – 2474.08	0.224
COVID.19 [Mild] *Antigen [NP Sf21]	1428.80	-5707.70 – 8565.30	0.694
COVID.19 [Moderate] *Antigen [NP Sf21]	2052.18	-4828.86 – 8933.21	0.558
COVID.19 [Severe] *Antigen [NP Sf21]	-7715.67	-14222.23 – -1209.12	**0.020**
COVID.19 [Mild] *Antigen [S1 Full HEK]	-645.71	-7782.21 – 6490.79	0.859
COVID.19 [Moderate] *Antigen [S1 Full HEK]	-8431.77	-15312.81 – -1550.74	**0.016**
COVID.19 [Severe] *Antigen [S1 Full HEK]	-15285.98	-21792.54 – -8779.43	**<0.001**
COVID.19 [Mild] * Antigen [S1 HEK]	4448.66	-2687.84 – 11585.16	0.221
COVID.19 [Moderate] *Antigen [S1 HEK]	-3845.54	-10726.58 – 3035.49	0.273
COVID.19 [Severe] *Antigen [S1 HEK]	-1546.90	-8053.46 – 4959.65	0.641
COVID.19 [Mild] *Antigen [S1 Sf21]	4188.98	-2947.52 – 11325.48	0.250
COVID.19 [Moderate] *Antigen [S1 Sf21]	3432.85	-3448.19 – 10313.88	0.328
COVID.19 [Severe] *Antigen [S1 Sf21]	10480.62	3974.07 – 16987.18	**0.002**
N_{id}	34		
Observations	714		
