# Supplementary material for: A new multiplex SARS-CoV-2 antigen microarray showed correlation of IgG, IgA, and IgM antibodies from patients with COVID-19 disease severity and maintenance of relative IgA and IgM antigen binding over time
Source: PLoS One. 2023 Mar 30;18(3):e0283537. doi: 10.1371/journal.pone.0283537 (PMC10062637; doi:10.1371/journal.pone.0283537)
Supplement: S8 Table — Significance of correlation analysis for first sampling point serum IgM samples binding to antigens with low intensity (3CLike Ecoli, Npro Full Ecoli, S1 Frag Ecoli, S2 Frag Ecoli, S2Pri Ecoli, and Spro Ecoli) compared to non-COVID-19 samples (NC). Bold p value indicates significant correlation. (PDF) [file pone.0283537.s016.pdf]

Table S8. Significance of correlation analysis for first sampling point serum IgM samples binding to antigens with low intensity (3CLike Ecoli, Npro Full Ecoli, S1 Frag Ecoli, S2 Frag Ecoli, S2Pri Ecoli, and Spro Ecoli) compared to non-COVID-19 samples (NC). Bold *p* value indicates significant correlation.

		**IgM**	
Predictors	*Estimates*	*CI*	*p*
(Intercept)	82.21	61.80 – 102.62	**<0.001**
COVID.19 [Mild]	46.65	14.57 – 78.73	**0.006**
COVID.19 [Moderate]	-31.78	-62.71 – -0.85	**0.044**
COVID.19 [Severe]	17.79	-11.47 – 47.04	0.224
Antigen [Npro Full Ecoli]	-53.19	-73.04 – -33.34	**<0.001**
Antigen [S1Frag Ecoli]	-60.68	-80.53 – -40.83	**<0.001**
Antigen [S2Frag Ecoli]	-47.51	-67.36 – -27.66	**<0.001**
Antigen [S2Pri Ecoli]	-37.66	-57.51 – -17.81	**<0.001**
Antigen [Spro Ecoli]	-61.98	-81.83 – -42.13	**<0.001**
COVID.19 [Mild] * Antigen [Npro Full Ecoli]	-67.02	-97.03 – -37.01	**<0.001**
COVID.19 [Moderate] *Antigen [Npro Full Ecoli]	15.23	-13.70 – 44.17	0.302
COVID.19 [Severe] *Antigen [Npro Full Ecoli]	-1.35	-28.71 – 26.01	0.923
COVID.19 [Mild] *Antigen [S1Frag Ecoli]	-8.29	-38.30 – 21.72	0.588
COVID.19 [Moderate] *Antigen [S1Frag Ecoli]	34.59	5.65 – 63.53	**0.019**
COVID.19 [Severe] *Antigen [S1Frag Ecoli]	11.96	-15.40 – 39.32	0.391
COVID.19 [Mild] * Antigen [S2Frag Ecoli]	-47.83	-77.84 – -17.82	**0.002**
COVID.19 [Moderate] *Antigen [S2Frag Ecoli]	37.51	8.57 – 66.45	**0.011**
COVID.19 [Severe] *Antigen [S2Frag Ecoli]	-19.80	-47.16 – 7.56	0.156
COVID.19 [Mild] * Antigen [S2Pri Ecoli]	-52.67	-82.69 – -22.66	**0.001**
COVID.19 [Moderate] *Antigen [S2Pri Ecoli]	38.44	9.50 – 67.37	**0.009**
COVID.19 [Severe] *Antigen [S2Pri Ecoli]	-17.13	-44.50 – 10.23	0.219
COVID.19 [Mild] * Antigen [Spro Ecoli]	-26.12	-56.13 – 3.89	0.088
COVID.19 [Moderate] *Antigen [Spro Ecoli]	36.00	7.06 – 64.94	**0.015**
COVID.19 [Severe] *Antigen [Spro Ecoli]	-4.09	-31.46 – 23.27	0.769
N_{id}	34		
Observations	612		
