# Supplementary material for: A new multiplex SARS-CoV-2 antigen microarray showed correlation of IgG, IgA, and IgM antibodies from patients with COVID-19 disease severity and maintenance of relative IgA and IgM antigen binding over time
Source: PLoS One. 2023 Mar 30;18(3):e0283537. doi: 10.1371/journal.pone.0283537 (PMC10062637; doi:10.1371/journal.pone.0283537)
Supplement: S9 Table — Significance of correlation analysis for first sampling point serum IgM samples binding to antigens with high intensity (B117 RBD HEK, NP Ecoli, NP HEK, NP Sf21, S1 Full HEK, S1 HEK, and S1 Sf21) compared to non-COVID-19 samples (NC). Bold p value indicates significant correlation. (PDF) [file pone.0283537.s017.pdf]

Table S9. Significance of correlation analysis for first sampling point serum IgM samples binding to antigens with high intensity (B117 RBD HEK, NP Ecoli, NP HEK, NP Sf21, S1 Full HEK, S1 HEK, and S1 Sf21) compared to non-COVID-19 samples (NC). Bold *p* value indicates significant correlation.

	IgM		
Predictors	*Estimates*	*CI*	*p*
(Intercept)	305.50	-617.69 – 1228.69	0.516
COVID.19 [Mild]	647.38	-804.29 – 2099.04	0.370
COVID.19 [Moderate]	1475.50	75.80 – 2875.20	**0.039**
COVID.19 [Severe]	1980.25	656.72 – 3303.77	**0.005**
Antigen [NP Ecoli]	3772.64	3062.98 – 4482.31	**<0.001**
Antigen [NP HEK]	266.87	-442.80 – 976.53	0.461
Antigen [NP Sf21]	-180.83	-890.49 – 528.84	0.617
Antigen [S1 Full HEK]	-217.19	-926.86 – 492.47	0.548
Antigen [S1 Hek]	-179.72	-889.39 – 529.95	0.619
Antigen [S1 Sf21]	-67.29	-776.95 – 642.38	0.852
COVID.19 [Mild] * Antigen [NP Ecoli]	-638.41	-1711.32 – 434.51	0.243
COVID.19 [Moderate] *Antigen [NP Ecoli]	-1286.73	-2321.24 – -252.23	**0.015**
COVID.19 [Severe] *Antigen [NP Ecoli]	-629.14	-1607.35 – 349.07	0.207
COVID.19 [Mild] * Antigen [NP HEK]	-479.05	-1551.97 – 593.86	0.381
COVID.19 [Moderate] *Antigen [NP HEK]	-1474.74	-2509.25 – -440.23	**0.005**
COVID.19 [Severe] *Antigen [NP HEK]	-214.26	-1192.47 – 763.94	0.667
COVID.19 [Mild] * Antigen [NP Sf21]	-653.36	-1726.28 – 419.55	0.232
COVID.19 [Moderate] *Antigen [NP Sf21]	-1231.12	-2265.63 – -196.62	**0.020**
COVID.19 [Severe] *Antigen [NP Sf21]	-1271.87	-2250.07 – -293.66	**0.011**
COVID.19 [Mild] * Antigen [S1 Full HEK]	-292.00	-1364.92 – 780.91	0.593
COVID.19 [Moderate] *Antigen [S1 Full HEK]	-1532.50	-2567.01 – -497.99	**0.004**
COVID.19 [Severe] *Antigen [S1 Full HEK]	-1772.80	-2751.01 – -794.59	**<0.001**
COVID.19 [Mild] * Antigen [S1 Hek]	203.68	-869.23 – 1276.60	0.709
COVID.19 [Moderate] *Antigen [S1 Hek]	-410.73	-1445.24 – 623.78	0.436
COVID.19 [Severe] *Antigen [S1 Hek]	-375.87	-1354.08 – 602.33	0.451
COVID.19 [Mild] * Antigen [S1 Sf21]	299.37	-773.55 – 1372.28	0.584
COVID.19 [Moderate] *Antigen [S1 Sf21]	16.73	-1017.78 – 1051.23	0.975
COVID.19 [Severe] *Antigen [S1 Sf21]	1549.98	571.77 – 2528.19	**0.002**
N_{id}	34		
Observations	714		
