# Supplementary material for: A new multiplex SARS-CoV-2 antigen microarray showed correlation of IgG, IgA, and IgM antibodies from patients with COVID-19 disease severity and maintenance of relative IgA and IgM antigen binding over time
Source: PLoS One. 2023 Mar 30;18(3):e0283537. doi: 10.1371/journal.pone.0283537 (PMC10062637; doi:10.1371/journal.pone.0283537)
Supplement: S10 Table — Biobank code, COVID-19 disease status of individual at sampling time, days post-symptoms onset at sampling time, biological sex (M, male; F, female), patient age at time of sampling, and serum IgG, IgA, and IgM concentration. N.d., not detectable; n.a., not assayed. (PDF) [file pone.0283537.s018.pdf]

Table S10. Serum sample information including follow up samples. Biobank code, COVID-19 disease status of individual at sampling time, days post-symptoms onset at sampling time, biological sex (M, male; F, female), patient age at time of sampling, and serum IgG, IgA, and IgM concentration. N.d., not detectable; n.a., not assayed.

Code	Disease	Days	Sex	Age	Serum Ig conc (mg/mL)		
					IgG	IgA	IgM
203-0041-1	Mild	19	M	80	32.16	47.21	1.86
203-0041-3	Mild	34	M	80	5.14	6.53	0.24
203-0077-1	Mild	25	M	62	3.26	6.07	0.09
203-0077-3	Mild	59	M	62	1.13	1.63	0.16
203-0054-1	Moderate	9	M	50	13.26	4.07	0.31
203-0054-2	Moderate	12	M	50	113.33	45.92	4.49
203-0004-1	Severe	18	M	42	51.30	25.68	2.78
203-0004-2	Severe	29	M	42	8.11	2.20	0.27
203-0015-2	Severe	16	M	69	75.25	31.57	4.83
203-0015-4	Severe	145	M	69	1.13	1.62	0.17
203-0018-1	Severe	19	M	66	30.53	20.31	0.71
203-0018-4	Severe	271	M	66	1.00	0.52	n.d.
203-0021-1	Severe	15	M	65	50.99	12.93	3.71
203-0021-2	Severe	17	M	65	56.13	2.89	0.35
203-0023-1	Severe	11	F	56	58.52	48.17	1.79
203-0023-3	Severe	33	F	56	28.49	7.73	0.46
203-0024-1	Severe	31	F	66	83.84	29.13	3.61
203-0024-2	Severe	34	F	66	n.a.	9.08	1.55
203-0078-2	Severe	9	F	35	23.08	8.30	1.80
203-0078-3	Severe	16	F	35	66.61	19.34	4.03
